# Laparoscopic application of intraoperative fascial traction (fasciotensⓇHernia) during loss of domain scrotal hernia repair: A European multicenter case series with technical details and preliminary results

**DOI:** 10.1007/s10029-026-03654-2

**Published:** 2026-03-17

**Authors:** Eva Barbosa, Gisella Barone, Camillo Leonardo Bertoglio, Matthias C. Schrempf, Metin Mazgaldzhi, Thomas Mones, Nihad Sardoschau, Fausto Catena, Fabio Cesare Campanile

**Affiliations:** 1 Complex Abdominal Wall Unit, General Surgery Department, Hospital de São João, Porto, Portugal; 2 Medical School of Porto University, Porto, Portugal; 3 Division of General Surgery, Magenta Hospital, ASST Ovest Milanese, Via Al Donatore Di Sangue, 51, 20013 Magenta (Mi), Italy; 4 Department of General, Visceral and Transplantation Surgery, University Hospital Augsburg, Augsburg, Germany; 5 Department of General &Amp, Visceral Surgery/Robotic Surgery, Augusta Hospital, Bochum, Germany; 6 General- and Visceral Surgery, Hernia Center, Maria-Hilf Hospital, Brilon, Germany; 7 Head of Abdominal Wall Surgery, Academic Teaching Hospital Püttlingen (Saarland University), Saarland, Germany; 8 Dept of Medical and Surgical Sciences Alma Mater, Studiorum University of Bologna, Bologna, Italy; 9 Bologna ITALY Dept of General and Emergency Surgery, Bufalini Hospital AUSL, Romagna, Cesena, Italy; 10 General Surgery Unit, Ospedale San Giovanni Decollato, Andosilla, Civita, Castellana, Italy

**Keywords:** Inguinoscrotal hernias, Loss of domain, Intraoperative vertical fascial traction

## Abstract

**Aim:**

To describe the laparoscopic intraoperative fascial traction (IFT) in the repair of scrotal hernia with loss of domain (LoD), focusing on the prevention of abdominal compartment syndrome (ACS).

**Methods:**

A multicenter retrospective analysis was conducted on nine consecutive patients with S2 and S3 LoD scrotal hernia, eligible for IFT, treated between November 2023 and August 2024 in eight European hospitals (Italy, Germany and Portugal). Technical details of laparoscopic IFT were documented. Postoperative intra-abdominal pressure (IAP), ventilatory parameters, complications, and recurrence were assessed.

**Results:**

The median Tanaka index was 0.57 and all patients underwent Lichtenstein repair; in two cases, a simultaneous preperitoneal mesh was added due to extensive inguinal defects. Median operative time was 210 min, with median IFT duration of 70 min and a traction force of 18 kg. Postoperative ACS did not occur. IAP was monitored in 55% of patients, with a median postoperative value of 11.4 mmHg. The median peak ventilation pressure before and after hernia reallocation was 16 and 19.5 mmHg respectively with a median differential of 3,5 mmHg (range 0–8). The median Intensive Care Unit (ICU) monitoring was 1 day, and the median hospital stay was 9.5 days. Five patients developed Clavien-Dindo grade I and II complications, with no recurrence detected after a median follow-up of 19 months.

**Conclusion:**

The laparoscopic IFT is a safe and useful adjunct in the surgical repair of LoD scrotal hernias. IFT may reduce the need for preoperative pneumoperitoneum and possibly prevent the development of postoperative ACS.

## Background

Inguinoscrotal hernias represent a distinct subset of inguinal hernias. The European Hernia Society (EHS) has introduced supplementary classification (S1-S3) and specific guidelines to deal with these patients [1]. Although they account for only about 6% of inguinal hernias in high income countries [1], the management of giant inguinoscrotal hernias (S2–S3) remains particularly challenging.

These hernias are usually long-standing, occur in patients with multiple comorbidities and can significantly impair quality of life, causing scrotal ulcers, voiding difficulties, mobility problems, bowel obstruction and low self-esteem [2].

Current evidence on the treatment of giant inguinoscrotal hernias, especially those with loss of domain (LoD) is scarce, consisting mainly of case reports [2–14]. As a result, no clear consensus has been established on the optimal preoperative strategy or surgical technique for these patients.

Several approaches have been described to restore the abdominal-visceral balance, including preoperative administration of Botulinum Toxin A (BTA) into the lateral abdominal wall, usually 4 weeks before surgery [15, 16]. Preoperative progressive pneumoperitoneum (PPP) is also often adopted to obtain a relevant gain of intraabdominal volume [17–20]. However, PPP can add morbidity and even mortality [21–23]. Other techniques, such as epiploon or viscera resection and component separation, have also been described to address the abdominal-visceral disproportion in giant scrotal hernias, with additional risks [2, 4, 6, 12].

Recently, intra-operative fascial traction (IFT) using the Fasciotens® device has emerged as a successful adjunct to achieve fascial closure in complex ventral hernia repair [24], reducing the need for more invasive procedures, decreasing morbidity. Building on these experiences, we hypothesize that IFT could also be applied intraoperatively in the treatment of LoD scrotal hernias. This technique may offer a reproducible and minimally invasive alternative, potentially reducing the need for PPP and minimizing the risk of postoperative abdominal compartment syndrome (ACS). This study details our method and preliminary outcomes.

## Methods

### Study design

This was a retrospective, multicenter case series. Data were collected from eight hospitals in three European countries (Italy, Germany, and Portugal) on patients operated between November 2023 and August 2024.

### Patient selection

**Fig. 1:**
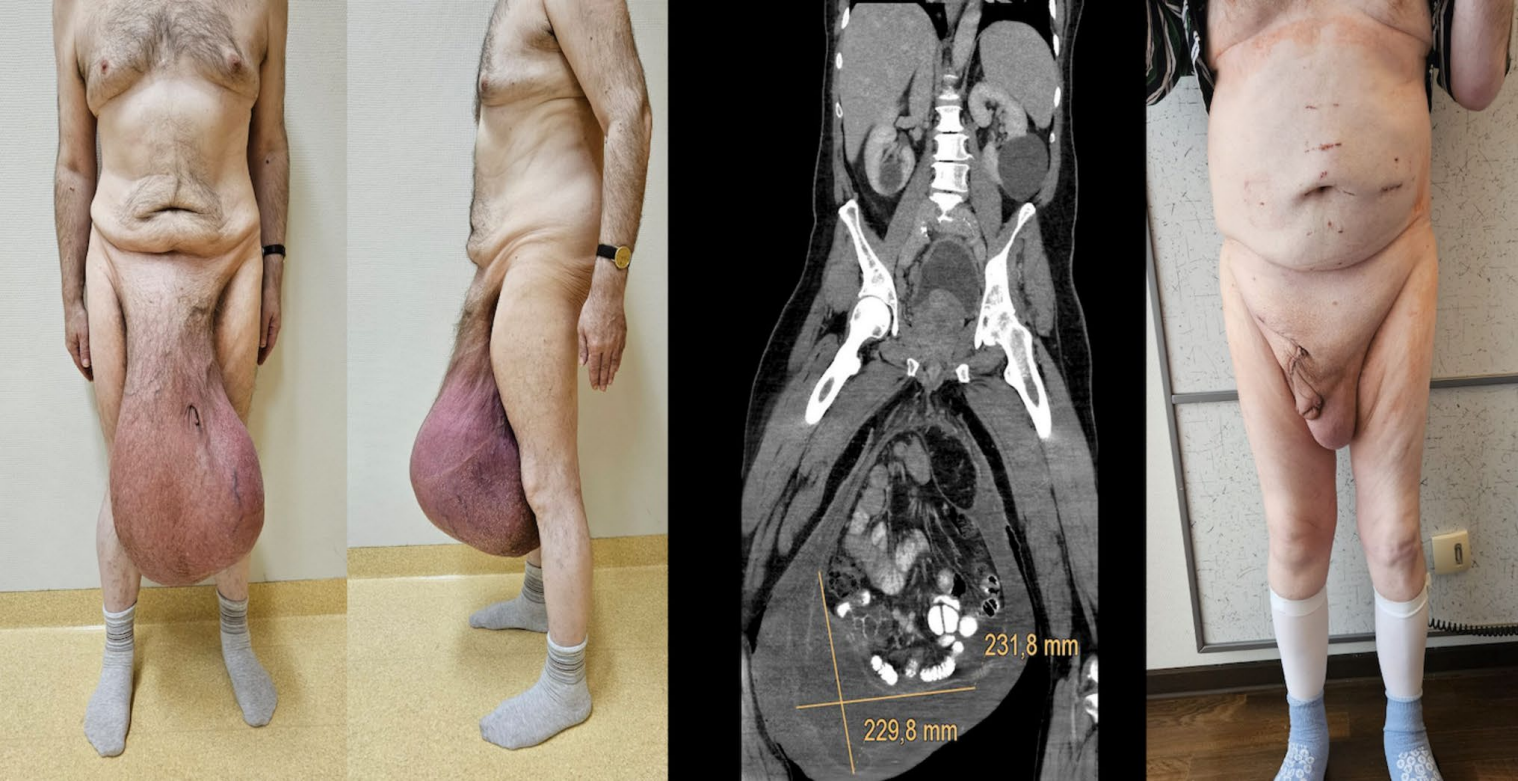
Illustrative patient of these series and its outcome

Patients with scrotal hernia classified as S2 or S3 (Fig. 1) according to the European Hernia Society supplementary classification, to whom was applied intraoperative fascial traction (IFT). Demographic data, comorbidities, hernia characteristics, prehabilitation strategies, intraoperative parameters, and postoperative outcomes were collected.

### Application of intra-operative fascial traction (Fig. 2)

Pneumoperitoneum was established either by Veress needle insertion at Palmer’s point or by mini laparotomy. The optical trocar was placed in the midline in the first two cases, but this position interfered with the placement of traction sutures. Hence, in subsequent cases, the optical trocar was placed along the umbilical transverse line in the right flank.

**Fig. 2:**
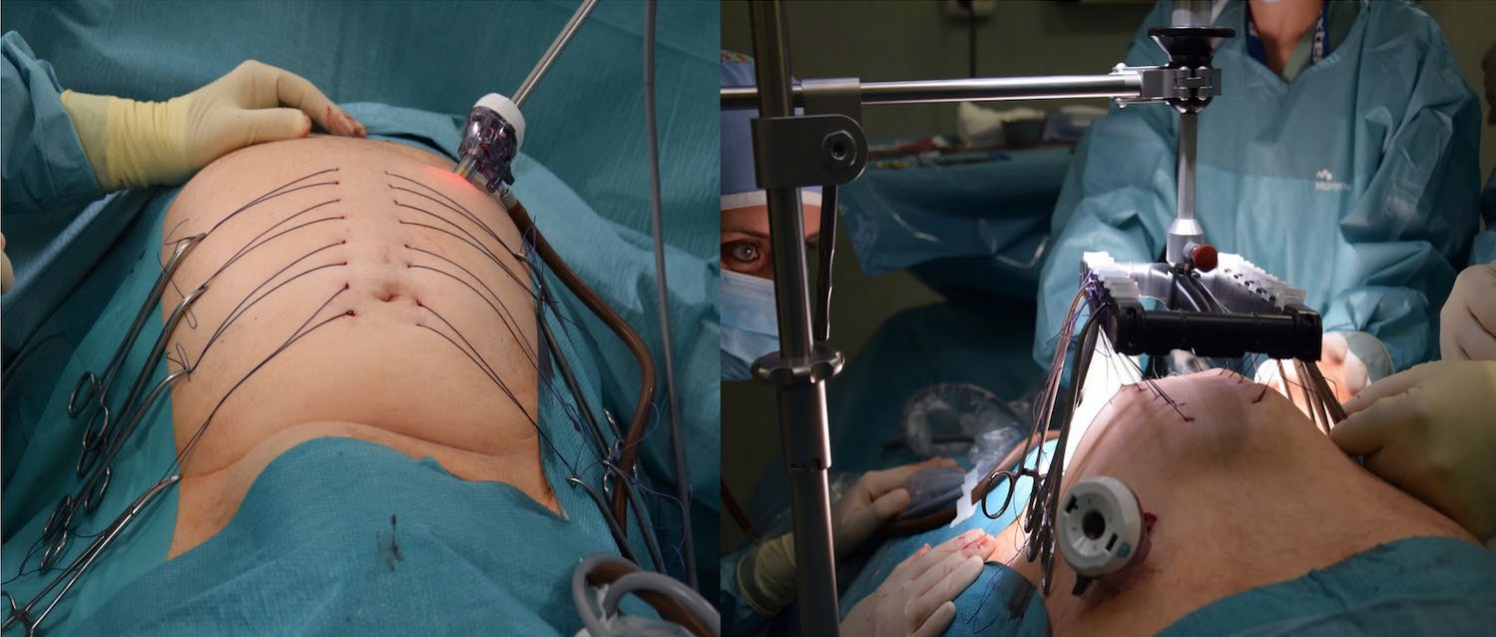
IFT assembly

Six U-sutures per side (Vicryl™ 2, Ethicon) were placed percutaneously, passing through the entire thickness of the abdominal wall, one centimeter lateral to the linea alba, using a suture passer. Each stitch was approximately 2 cm in length, and the U-sutures were equally distributed from cranial to caudal. After placement, the pneumoperitoneum was released, the Fasciotens®Carrier and Fasciotens®Hernia were assembled, and the traction sutures attached to the suture retention frame.

IFT was initiated with a traction force of approximately 14 kg, progressively increased to a maximum of 18 kg and then maintained during the procedure. Traction duration was adapted intraoperatively according to hernia reduction and physiological tolerance, rather than following a predefined time protocol. During the traction time, the inguinal hernioplasty was performed with mesh positioning, according to the surgeon’s preference.

## Results

The main patient's demographic characteristics are shown on Table 1. At the time of surgery, the median age was 71 years (range 60–87), and the median BMI 27 kg/m^2^. The most common comorbidities were hypertension (5 patients), diabetes (4 patients), and cardiovascular disease (3 patients). Two patients reported nicotine or cocaine use. All patients underwent abdominal wall prehabilitation with botulinum toxin type A (BTA) 3–5 weeks before surgery. Doses ranged from 200 to 1000 IU. Each center used a single formulation—either Botox® or Dysport®—administered bilaterally, according to institutional protocols. Two patients underwent progressive preoperative pneumoperitoneum (PPP) 7 days before surgery, with a mean insufflated volume of 1750 ml. PPP was discontinued in one patient because of catheter damage on day 2 and in the other due to preferential scrotal air inflation. In both cases, the strategy was converted from PPP to IFT.

[Edit]

**Table 1:** Patients Characteristics RD, Rectus Diastasis; BMI, Body Mass Index; EHS, European Hernia Society; ASA, American Society of Anesthesiology; BTA, Botulinum Toxin A; PPP, Progressive Preoperative Pneumoperitoneum; IU, International Unit

Patient Characteristics	
Median age, years (Range)	71 (60–87)
Median ASA score (Range)	3 (2–3)
Median BMI, kg/m^2^ (Range)	27 (23–35)
Comorbidities/Risk factors	
Diabetes	4
Hypertension	5
Cardiovascular diseases	3
Renal diseases	1
Liver diseases	1
Pulmonary diseases	1
Previous oncological diseases	1
Obesity, mild	2
Smoke	1
Cocaine abuse	1
Hernia Characteristics	
Side	
Unilateral	5
Bilateral	4
Primitive	8
Recurrent	1
EHS Classification	
S1	-
S2	4
S3	4
Median Tanaka Index (Range)	0.57 (0.2–0.8)
Association with RD	1
Previous surgery	4
Abdominal wall prehabilitation	
BTA	9
200 IU (Botox®)	2
300 IU (Botox®)	3
500 IU (Dysport®)	3
1000 IU (Dysport®)	1
PPP (1500—2000 mL)	2

Intraoperative and postoperative data are shown in Tables 2 and 3.

**Table 2:** Intraoperative data. IFT, Intraoperative Fascial Traction

*Intraoperative Data*	
Median operative time, min	210
(Range)	(126–330)
Median operative time for unilateral hernia, min	176
(Range)	(126–215)
Median operative time for bilateral hernia, min	280.5
(Range)	(192–330)
Median IFT time, min	70
(Range)	(40–180)
Median IFT Traction, kg	18
(Range)	(14–18)
Mesh Type	
Polypropylene	5
+ Polyglecapron	1
PVDF	2
Polyester	1
Mesh Size, cm	
6 × 13.7	1
7.5 × 12	1
7.5 × 15	2
8 × 12	1
9 × 15	1
10 × 15	1
25 × 20	1
25 × 30	1
Kind of repair	
Lichtenstein	7
(Uni/Bilateral)	
Double repair (open anterior preperitoneal repair with posterior inguinal wall reconstruction + Lichtenstein)	2
Other surgical procedure	
Orchiectomy	1
Scrotal remodeling	1
Median Ventilation peak pressure *before* repositioning of hernia, mmHg	16
(Range)	(10—22)
Median Ventilation peak pressure *after* repositioning of hernia, mmHg	19.5
(Range)	(10—22)
Median Differential of peak pressure, mmHg	3.5
(Range)	(0–8)

**Table 3:** Postoperative data ICU, Intensive Care Unit. IAP, Intra-Abdominal Pressure. C-D, Claviend-Dindo. SSO/SSI, Surgical Site Occurrences/Surgical Site Infections

*Postoperative Data*	
ICU Recovery	
No	3
Yes	6
Median ICU Recovery, days	1
(Range)	(1–3)
Median Postoperative IAP, cmH20 (Range)	11.4 (8—25)
Median Hospital stay, days (Range)	9.5 (2—28)
Complications, C-D grade	
No complications	4
I	3
II	1
III	1
IV	-
30 days surgical site occurrences/infections	
SSO	3
SSI	1
Median follow-up, months	19
(Range)	(13–22)

**Fig. 3:**
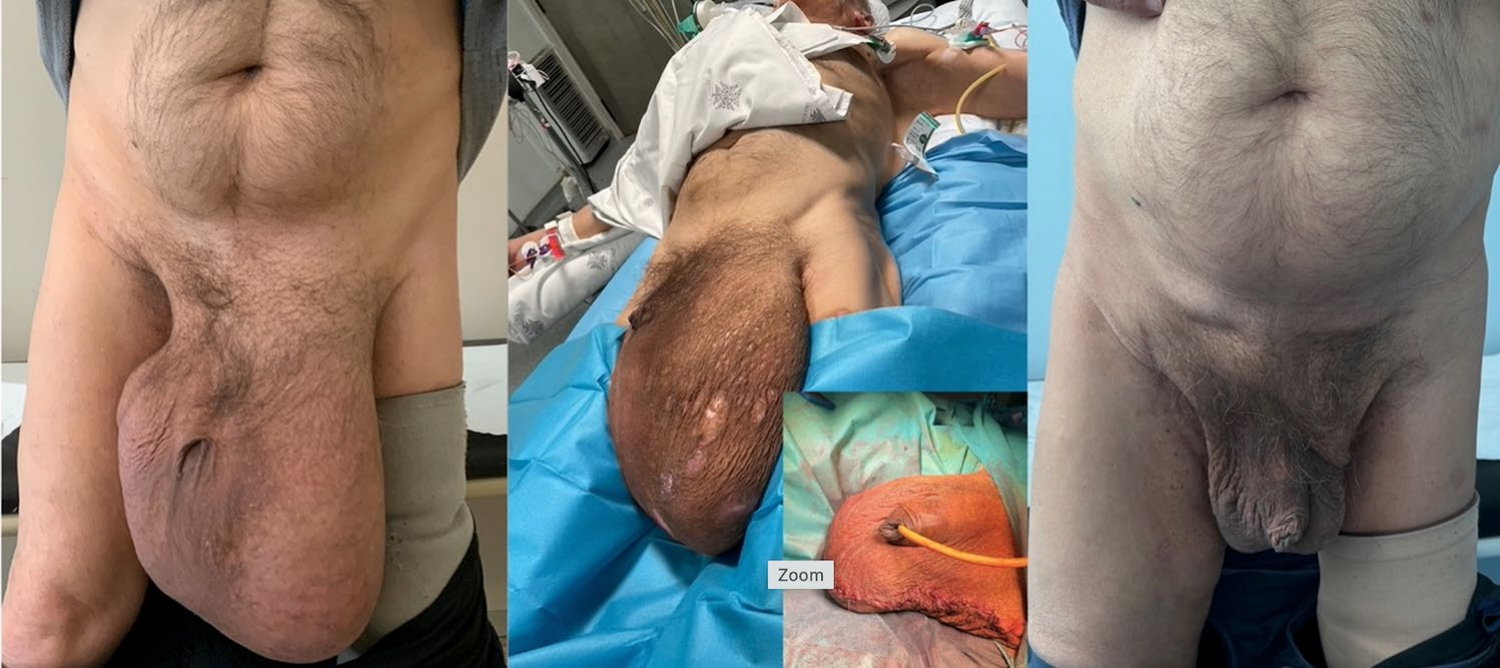
S3 inguinoscrotal hernia with Lod. Skin ulcers in the scrotum. Scrotal remodeling at the end of the hernia surgery. Postoperative outcome at follow up

The median operative time was 210 min (176 min for unilateral and 285.5 min for bilateral). Median IFT time was 70 min (range 40–180), with a maximum traction force of 18 kg. Lichtenstein repair was performed in seven patients, while two required additional preperitoneal mesh reinforcement due to extensive posterior wall defects. A polypropylene mesh was used in five patients, in one case associated with polyglecapron. The mesh dimensions varied among patients but with a minimum of 7 × 12 cm. Scrotal remodeling was necessary in one of the patients due to scrotal ulcers (Fig. 3). One patient underwent an associated right orchiectomy due to prior testicular gangrene.

Six patients (66%) required initial ICU monitoring with a median stay of 1 day. The IAP was measured after the reduction of hernia content in five patients, with a median value of 11.4 mmHg; one patient reached a 25 mmHg on postoperative day 1, which resolved conservatively without any signs of ACS (abdominal compartment syndrome). No ACS was reported postoperatively. The median overall hospital stay was 9.5 days (range 2–28). Postoperative complications occurred in five patients, most of them Clavien–Dindo grade I–II. Reported events included scrotal wound dehiscence, scrotal hematoseromas (two cases, one requiring revision), one superficial surgical site infection treated with open wound and negative pressure wound therapy (NPWT) (Suprasorb® CNP,Lohmann&Rauscher), and one respiratory infection treated with antibiotics.

At a median follow-up of 19 months (range 13–22), no hernia recurrences were detected.

## Discussion

This study presents the first multicenter case series with IFT in the management of giant inguinoscrotal hernias with LoD. It is also one of the largest in the literature with quantification of the LoD in giant inguinoscrotal hernias. The main findings are that laparoscopic IFT was applied safely across different surgical centers, enabled reduction of hernia contents without clinical postoperative ACS, avoiding the need for more invasive strategies such as component separation or visceral resections. Postoperative morbidity was acceptable and limited mainly to minor complications, and no recurrences were observed during the follow-up period.

The strengths of this study include its multicenter design, which shows that the laparoscopic IFT technique can be adopted in different environments, and the systematic quantification of LoD, which is rare in the literature on giant inguinoscrotal hernias. Moreover, all patients underwent some form of prehabilitation, providing a realistic view of multimodal management in this setting.

However, important limitations must be acknowledged. Despite being multicentric, the series includes only nine patients, reflecting the rarity of this condition but limiting statistical power. The retrospective design and inter-center heterogeneity in prehabilitation protocols, BTA use, PPP application, and IAP measurement limit the strength of the conclusions. In addition, operative times were not recorded in a sufficiently granular manner to allow separation between IFT set-up and hernia repair time, and no formal inter-center outcome comparison, learning curve assessment, or technique standardization metrics could be performed.

LoD is associated with serious risks when large volumes of viscera are returned to the abdominal cavity, including ACS and death [7, 25]. The definition LoD remains debated [26, 27]. Conventionally, LoD is quantified using volumetric indices: the Tanaka index (hernia sac volume/abdominal cavity volume ≥ 0.25 [28] and the Sabbagh index (hernia sac volume/peritoneal volume) ≥ 20% [29]. Importantly, Tanaka himself specified that the 25% threshold was chosen arbitrarily to allow case standardization rather than representing a biologically validated cut-off. All but one patient fulfilled the ≥ 0.25 Tanaka criterion, despite its acknowledged arbitrariness.

Prehabilitation and pre-operative adjuvants such as weight loss, BTA, and PPP, are usually essential to achieve a good outcome in cases of LoD. A recent systematic review identified only 81 reported patients with giant inguinoscrotal hernias [19], where LoD was managed with BTA + PPP in 80% of the cases, PPP alone in 10% and BTA alone in the remain 10%. In our cohort, BTA was administered in all patients, consistent with the evidence that BTA increases lateral abdominal wall compliance [30]. Nevertheless, the different institutional BTA protocols used is an important limitation in this study. PPP was used in only two patients, with limited insufflation volumes, and was discontinued early in one case due to catheter disruption and in another due to gas leakage into the scrotum. We believe that the volumes of air introduced (1550-2000 ml) were not sufficient to make a difference in the LoD management and in the post-operative outcome, especially because a considerable part of the insufflated air tends to migrate to the inguinal hernia sac. Nevertheless, the heterogeneity in prehabilitation strategies reflects real-world practice and precludes isolation of the independent effect of any single adjunct, including IFT.

Compared with PPP, which has reported adverse events in up to 12% of patients, including pain, dyspnea, thromboembolic events, subcutaneous emphysema, pneumomediastinum, pneumopericardium, infection, abscess, and visceral puncture, and even mortality in rare cases [23], the IFT in our series was performed without direct complications. In addition, in many centers, a hospital admission is necessary during the insufflation periods of PPP, adding costs to the procedure and changes in patients’ lives. These highlight both the limitations of PPP and the potential role of IFT as an alternative strategy.

Regarding the surgical technique, the preference for the Lichtenstein repair reflects existing evidence. In a systematic review, 70% of giant inguinoscrotal hernias were repaired through an anterior approach, with Lichtenstein accounting for 38%, followed by Stoppa and transabdominal preperitoneal repairs [19]. Our findings therefore align with the broader literature, although longer follow-up is needed to confirm durability. None of the patients in this study needed extensive surgery such as component separation or visceral resections in order to deal with the LoD as in other reports.

There were no deaths in our group, and postoperative complications were mainly Clavien-Dindo I and II which is consistent with the literature for treatment of giant inguinoscrotal hernias, although a systematic review reported four deaths, one of which was due to ACS [31]. In our series only one patient had a postoperative IAP of 25 mmHg, without clinical abdominal compartment syndrome and resolved with conservative measures.

There are no recurrences after a median follow-up of 19 months, in line with the low recurrence stated in the literature for these giant hernias [31].

The findings of this study suggest that laparoscopic IFT may be a valuable adjunct within a multimodal strategy for the management of giant inguinoscrotal hernias with LoD. By enabling controlled intraoperative expansion of the abdominal cavity, IFT may mitigate the risk of postoperative ACS and reduce reliance on preoperative PPP, which is resource-intensive, uncomfortable for patients, and not without risks. The association of IFT with BTA, used in all our cases, could provide a synergistic effect by increasing abdominal wall compliance and facilitating safe hernia reduction with a durable elongation of the lateral wall, thus preventing postoperative IAH and ACS. For clinicians, this approach offers a minimally invasive option that may simplify management of these rare but complex cases.

Despite promising results, unanswered questions remain. The long-term recurrence rate after IFT-assisted repair is unknown and requires studies with longer follow-up. The optimal prehabilitation strategy—whether BTA alone, BTA plus IFT, or IFT combined with PPP—remains to be defined. In addition, the cost-effectiveness of IFT compared with PPP and other strategies has yet to be established. Future prospective multicenter studies with standardized protocols, structured time recording, and inter-center outcome analysis are necessary to validate these preliminary findings and to better define the role of laparoscopic IFT in the treatment algorithm of giant inguinoscrotal hernias with LoD.

## Conclusions

This multicenter case series describes the laparoscopic application of intraoperative fascial traction (IFT) in the repair of giant inguinoscrotal hernias with loss of domain. The technique proved safe across different centers, with no intraoperative complications, no postoperative clinical established abdominal compartment syndrome, and acceptable morbidity in a high-risk population.

These preliminary findings suggest that laparoscopic IFT may represent a valuable adjunct in the surgical management of giant scrotal hernias with LoD, particularly when combined with botulinum toxin A. Nevertheless, the retrospective nature of the study and the small sample size preclude definitive conclusions regarding the ability of IFT to safely prevent abdominal compartment syndrome. Larger prospective studies using standardized protocols are needed to better define its role within treatment algorithms.
